# Evaluation of transformer models for financial targeted sentiment analysis in Spanish

**DOI:** 10.7717/peerj-cs.1377

**Published:** 2023-05-09

**Authors:** Ronghao Pan, José Antonio García-Díaz, Francisco Garcia-Sanchez, Rafael Valencia-García

**Affiliations:** Faculdad de Informática, Universidad de Murcia, Murcia, Spain

**Keywords:** Sentiment analysis, Natural language processing, Financial domain, Targeted sentiment analysis

## Abstract

Nowadays, financial data from social media plays an important role to predict the stock market. However, the exponential growth of financial information and the different polarities of sentiment that other sectors or stakeholders may have on the same information has led to the need for new technologies that automatically collect and classify large volumes of information quickly and easily for each stakeholder. In this scenario, we conduct a targeted sentiment analysis that can automatically extract the main economic target from financial texts and obtain the polarity of a text towards such main economic target, other companies and society in general. To this end, we have compiled a novel *corpus* of financial tweets and news headlines in Spanish, constituting a valuable resource for the Spanish-focused research community. In addition, we have carried out a performance comparison of different Spanish-specific large language models, with MarIA and BETO achieving the best results. Our best result has an overall performance of 76.04%, 74.16%, and 68.07% in macro F1-score for the sentiment classification towards the main economic target, society, and other companies, respectively, and an accuracy of 69.74% for target detection. We have also evaluated the performance of multi-label classification models in this context and obtained a performance of 71.13%.

## Introduction

In the past, managing financial data was limited to specific applications for banks and financial companies. However, since the emergence of Web 2.0., social networks, Internet of Things (IoT) devices and the popularity of wearable devices such as smartphones or smartwatches, there are vast amounts of economic, financial and monetary data publicly available in websites and social networks. Such data can be automatically analyzed and leveraged to monitor public opinion, receive early warnings, and perform positive and negative impact analysis (Paul et al., 2016). Moreover, the rise of modern large language models (LLMs) based on Transformers (Kalyan, Rajasekharan & Sangeetha, 2021) has enabled an increase in the performance of financial tasks such as risk assessment (Yang, Yuan & Lau, 2022) or stock market analysis (Sonkiya, Bajpai & Bansal, 2021), just to name but a few.

Sentiment analysis (SA) is a popular natural language processing (NLP) task that can be applied to the financial domain. In brief, SA consists in automatically determining if a piece of text contains a positive, neutral or negative emotion. SA has proven effective in the field of economics (Goodell et al., 2023). However, financial information is complex and the semantic ambiguity in these texts is quite abundant and very difficult to manage (Salas-Zárate et al., 2017b). The identified challenges are concerning the complexity of the financial language and the different point of views in which the polarity can be classified. First, financial language is inherently complex since financial terms refer to an underlying social, economic and legal context (Milne & Chisholm, 2013). Second, financial language is highly dependent on the context, since an expression may have either positive or negative connotations depending on the context where it is used. For example, the expressions “Rising electricity price” and “Rising salaries” both contain the word “rising” but the subjective polarity of both is different. Third, the identification of the main economic target (MET) to which the sentiment is referring to is complex to determine because there might exist several possible candidates or may not be explicitly stated in the text. Fourth, the overall sentiment within the financial domain is hard to establish, because an event can be deemed positive or negative if considering other targets apart from the MET. For instance, there are financial news which are positive for banks but negative for the citizens or for any other business sector. Fifth, the large amount of novel LLMs based on Transformers available (Kalyan, Rajasekharan & Sangeetha, 2021) makes it difficult to know beforehand which model is best suited for the financial domain.

The contributions of this work to the field of SA for the financial domain are as follows. First, the release of the Spanish FinancES-TSA 2022 *corpus*, with 3,829 financial headlines and micro-blog posts that have been manually annotated with the MET and three subjective polarities in a three-level range (*i.e*., positive, negative or neutral) towards three targets: MET, other companies, and the society. Second, we propose a new financial targeted SA method, trained with the Spanish FinancES-TSA 2022 *corpus*, that is able to extract the MET from a text and determine whether the sentiment is positive, negative, or neutral with respect to the aforementioned three targets. The presented method is based on supervised deep learning with LLMs, which are used for both tasks, namely, MET extraction and targeted SA. Third, several Spanish LLMs models with different architectures are evaluated for their applicability to the financial domain. These models are evaluated both individually and combined by means of ensemble learning. Fourth, two schemes for training the sentiment classifiers are compared. On the one hand, the training of one classifier per target and, on the other, a multi-label scheme in which all sentiments for each target are trained at the same time.

This article is structured as follows. Section 2 includes the state of the art of SA and its application to the financial domain. The materials and methods used are described in Section 3, which includes the pipeline of our proposal and details about the *corpus* compilation and annotation process. Section 4 presents the experiments carried out to evaluate the different models considered (*i.e*., BETO, MarIA, BERTIN, ALBETO, and DistilBETO) and discusses the results. Finally, in Section 5 conclusions and future work are put forward.

## State-of-the-art

In this section, a summary of the state-of-the-art and literature review of studies focusing on the use of natural language processing (NLP) techniques for SA (see “Natural language processing for sentiment analysis”) and their application in the financial domain (see “Sentiment analysis in the financial domain”) are presented.

### Natural language processing for sentiment analysis

LLMs have resulted in one of the major advances in Artificial Intelligence (AI) (Veres, 2022). LLMs are general-purpose models that are trained using gigantic datasets that can be easily adapted to several NLP tasks including natural language generation, summarization, translation or classification. LLMs are based on two pillars: attention and transfer learning. The attention mechanism (Vaswani et al., 2017) allows obtaining embeddings (*i.e*., language units such as words can be represented as dense vectors with semantically similar units having similar representation) that are context-aware; that is, the representation of the same linguistic unit varies according to the surrounding words. Attention solves important linguistic problems related to polysemy and word disambiguation and it allows the model to generate or understand large and coherent text. Transfer learning (Bozinovski, 2020), on the other hand, enables LLMs to be adapted for solving specific tasks; that is, they provide general-purpose language understanding capabilities that can be fine-tuned to solve tasks in other domains in which the language or the background information is more precise. Some of the first and most popular LLMs are BERT (Devlin et al., 2019), RoBERTA (Liu et al., 2019) or ALBERT (Chiang, Huang & Lee, 2020). The original version of these models was available only for the English language; however, multi-language variants (Conneau et al., 2020) or LLMs trained for languages other than English, like Spanish (Gutiérrez-Fandiño et al., 2022; Cañete et al., 2020; de la Rosa et al., 2022), soon appeared.

One of the main disadvantages of LLMs is that they are computationally expensive. In fact, the usage of these models is not reasonable in computers without GPUs or TPUs, neither during training nor during inference. Therefore, some techniques are focused on simplifying LLMs by means of distillation and quantization. DistilBERT (Sanh et al., 2019), for instance, is a lightweight version of BERT trained using distillation. Another disadvantage of LLMs is that they are black-box models, so their outputs are hard to interpret. In this sense, it is possible to complement LLMs with other feature sets, such as linguistic features, that are easier to interpret (García-Díaz, Colomo-Palacios & Valencia-García, 2022). Some strategies for combining LLMs and other feature sets are (i) ensemble learning (Rokach, 2010), in which the predictions of each model and feature are combined to build more robust models; or (ii) mixture of experts (Du et al., 2022), which relies on the divide-and-conquer principle splitting the problem space, covering different input regions with different learners.

The analysis of textual sentiment is concerned with the categorization of the opinions expressed in a text, *i.e*., extracting and analyzing subjective information about the polarity of a text (Pang & Lee, 2008). This polarity can be expressed in the form of a range, from a binary classification (‘positive’ or ‘negative’) to a more complex one (*e.g*., ‘very negative’, ‘negative’, ‘neutral’, ‘positive’, or ‘very positive’). Three levels of analysis depending on the degree of specificity can be distinguished, namely, document-based, sentence-based and aspect-based (ABSA) (Ligthart, Catal & Tekinerdogan, 2021). Document-based SA assumes that each document expresses only one main sentiment. With sentence-based SA, a sentiment is calculated for each sentence in the document. ABSA divides texts into subtopics and assigns a sentiment to each one, thus becoming the most sophisticated approach for conducting SA. Brauwers & Frasincar (2023) provide a comprehensive survey on ABSA. In their state-of-the-art study, the authors discuss models based on Transformers along with hybrid deep learning models that incorporate dictionary-based, ontology-based or discourse-based knowledge bases. The survey concludes that while adopting known datasets enables comparative analyses, other domains beyond restaurant reviews, electronics reviews and Twitter data should be explored. The authors also highlight the importance of developing datasets for languages other than English.

There are top academics and researchers working on SA from Spanish texts, with relevant results in the last few years. A SA model for Spanish tweets is proposed in García-Díaz et al. (2023). A flexible feature extraction method has been conceived in which feature selection is customized to each word depending on its context. Two corpora of Spanish tweets have been used for training and evaluation. The proposed neighbor-sentiment algorithm resulted in improvements of both F1-score and accuracy over previous works. Martínez-Seis et al. (2022) describe a deep learning approach for ABSA of restaurants reviews in Spanish. The architecture presented is composed of two convolutional neural networks (CNN), one devoted to polarity identification and the other responsible for the aspect-based sentiment classification. The Spanish subset of the *corpus* of restaurant reviews available from the 2016 edition of the SemEval competition was used in the experiments. The aspect extraction approach described in the work showed an improvement on the results of previous work, but the accuracy of their sentiment classification system is rather low. To conclude this SA overview, in a recent review, Osorio-Angel, Peña-Pérez-Negrón & Espinoza-Valdez (2021) studied the latest advances in SA for the Spanish language. While the pipeline and the techniques used at each step (information extraction, preprocessing, feature extraction, sentiment classification and evaluation) are comparable to those used for any other language, the authors highlight the ever-growing number of linguistic resources (*e.g*., lexicon or *corpus*) explicitly developed for the Spanish language. In terms of performance, deep learning models achieved the best results.

### Sentiment analysis in the financial domain

In the field of finances, the purpose of SA is to capture investors’ sentiments and emotions towards the financial market expressed in social networks or media and to quantify these emotions as numerical variables that will potentially be predictors of the stock market (Arratia-Quesada, 2021; Nemes & Kiss, 2021; Goodell et al., 2023). However, different factors hamper the effectiveness of SA in the financial domain. First, financial language is inherently complex since financial terms refer to an underlying social, economic and legal context (Milne & Chisholm, 2013). Furthermore, the type of language used within this domain is highly dependent on the context since one word or expression may have either positive or negative connotations depending on the context where it is used (stock market shares rise, debt rises, *etc*.). Finally, the degree of subjectivity within the financial domain is hard to determine, because an event can be deemed positive or negative depending on the point of view. For instance, there are financial news which are positive for banks and very negative for any other business sector and for society in general.

One of the first publications related to SA of financial texts is (Tetlock, 2007), which published a set of lexicon-based analysis techniques based on BoW (Bag of Words) to classify implicit sentiments in financial news. This technique consists of constructing a list of words, where each of these lists and labels is associated with a positive or negative category. Using a classification based on word categories drawn from the *Harvard Psychosocial Dictionary*, Tetlock (2007) quantified the optimism and pessimism contained in the Wall Street Journal’s “Abreast of the Market” column and found that high levels of pessimism reflected by the news as a whole predicted market price declines.

One of the main problems related to financial analysis, and more specifically to the stock market, is that current approaches have not been designed to take into account the sentiments from different viewpoints, which might be relevant to support investment decisions on companies or business sectors. In recent years, some studies have emerged on the relationship between public sentiment and stock prices (Li et al., 2014). However, due to the complexity of the financial domain, the outcome of the proposed approaches is not good enough (Kharde & Sonawane, 2016).

Othan, Kilimci & Uysal (2019) used a deep neural network and a BERT transformer to predict the direction of stock prices in the Turkish stock market (BIST100) by employing Turkish texts from social media platforms. Salas-Zárate et al. (2017b) presented a feature-based opinion mining method. Their work is based on a semantic orientation approach to detect polarity. In particular, they used the SentiWordNet lexicon and N-gram methods. In a further study successful results were obtained by evaluating several Spanish Transformers to detect the polarity of financial tweets (García-Díaz, García-Sánchez & Valencia-García, 2023). The method used a set of lexico-morphological and semantic features.

In previous works, our research group has explored different approaches for SA from Spanish texts in various application domains (Salas-Zárate et al., 2014; Peñalver-Martínez et al., 2014; Salas-Zárate et al., 2017a; Paredes-Valverde et al., 2017; García-Díaz, García-Sánchez & Valencia-García, 2023). On the basis of the analysis presented above, our work has some relevant and unique aspects. First, we have compiled a dataset of financial tweets and headlines in Spanish, which represents an important resource for the research community focused on this language. The *corpus* has been manually annotated with the MET and the sentiment polarity from three perspectives: the MET, other companies, and society in general. Second, we propose a targeted SA method that extracts the MET from textual content in financial texts and then obtains the sentiment polarity of the text towards the target and the other economic agents involved, namely, other companies and society in general. Besides, we evaluate the performance of a number of state-of-the-art LLMs including BETO, MarIA, BERTIN, ALBETO, and DistilBETO, and the impact of combining the predictions of the models through ensemble learning is assessed.

## Materials and Methods

**Figure 1:**
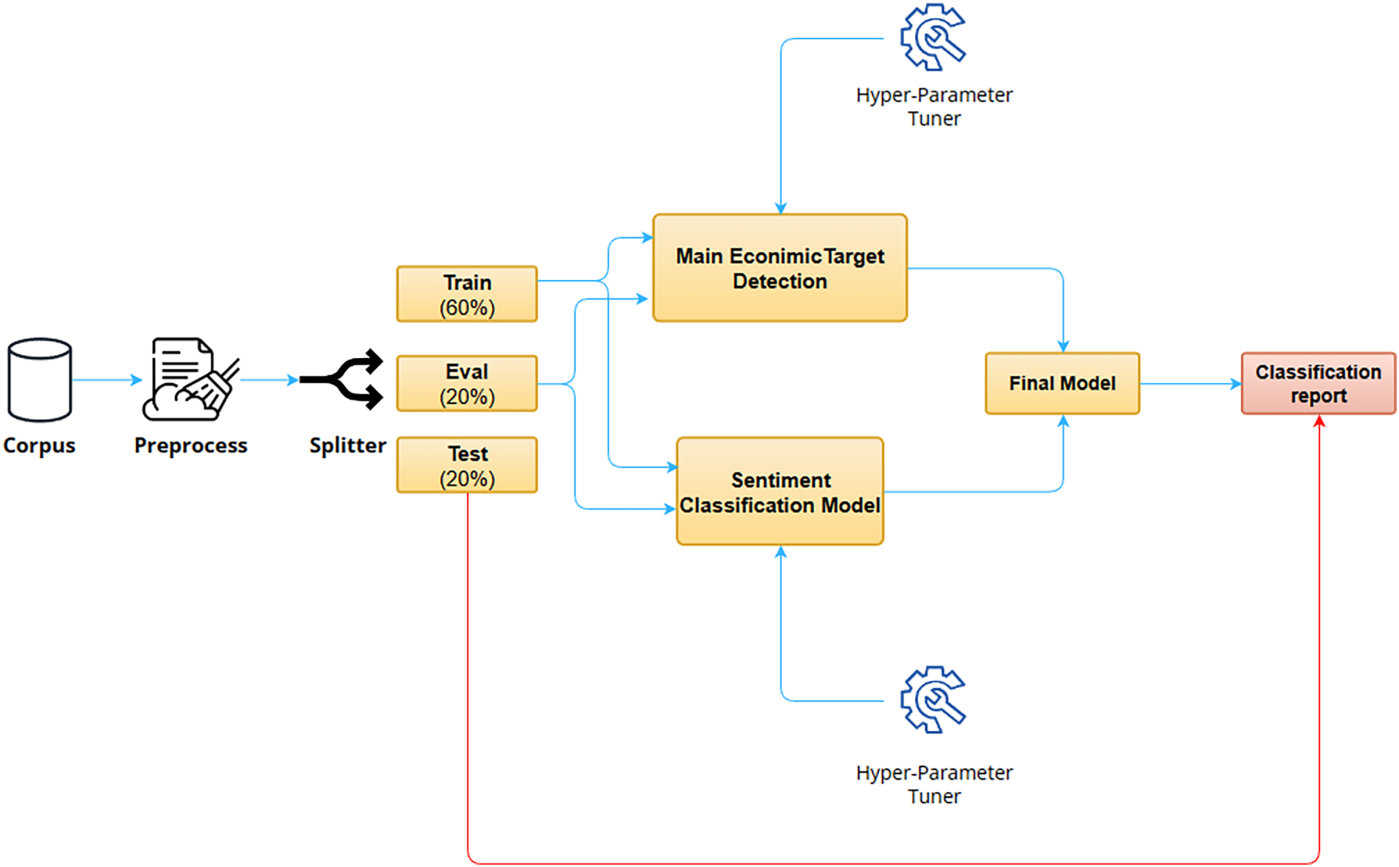
Overall architecture of the financial targeted sentiment analysis system for Spanish texts.

In this section a summary of the materials, methods, proposed framework, and LLMs is presented. Figure 1 depicts the pipeline of this work. In a nutshell, the process is as follows. The input of the system is a novel *corpus* that has been compiled for targeted SA for the financial domain in Spanish. This *corpus* was extracted from two data sources: headlines from news sites and micro-blogging posts. First, the *corpus* is pre-processed to normalize the texts from the different data sources. Second, the *corpus* is divided into training, validation and testing. Train and validation splits are then used to train with hyperparameter evaluation the two main modules of the system, namely, a named-entity recognition (NER) model for the extraction of the MET and a sentiment classification model for obtaining the sentiments toward the targets under question (MET, other companies, and society in general). The training of these models involves the evaluation of several Spanish LLMs and their combination by means of ensemble learning. Finally, the results are evaluated to obtain the best system. Next, extensive details are provided about the *corpus* that has been created and the different stages that comprise the proposed pipeline.

### Datasets

As far as we are concerned, there are no available corpora for targeted SA in Spanish focused on the financial domain. Consequently, a novel *corpus* has been compiled and annotated for this task. Two data sources were selected to compile the *corpus*: (i) micro-blogging posts from different financial accounts, and (ii) financial headlines from news sites. Both data sources have been combined to produce the Spanish FinancES-TSA 2022 *corpus*. In the following, some additional details about are given about these data sources.

Twitter is a micro-blogging platform and a reference data source for NLP given that its content is public and the limit in the maximum length of the tweets favors the conciseness and directness of the text. A clear example of how a tweet can affect or predict the behavior of the financial market is the case of *DogeCoin*, which experienced a 26% rise after a post by Elon Musk on his Twitter account (https://es-us.finanzas.yahoo.com/news/dogecoin-elon-musk-twitter-132158868.html). Tweepy (https://github.com/tweepy/tweepy) has been used to compile tweets from eight official Twitter accounts from news sites with financial information in Spanish: *@expansioncom*, *@elpais_economia*, *@elEconomistaes*, *@CincoDiascom*, *@bolsamania*, *@Invertia*, *@abceconomia*, and *@Modaes*. These were selected because they are accounts from digital newspapers (we did not want to include personal accounts that include personal opinions) and these media sites publish new content daily. In the first stage, a total of 3,829 tweets were extracted. We discarded those tweets that did not explicitly state the MET to which the sentiment refers, resulting in a final dataset with 1,563 Spanish financial tweets.

The second data source are headlines written in Spanish from digital newspapers focused on financial content. We selected a total of 92 newspapers, including Expansión (https://www.expansion.com/), El Economista (https://www.eleconomista.es/) or modaES (https://www.modaes.es/), among others. It is worth mentioning that the newspapers are from different Spanish-speaking countries, not just from Spain. To compile this dataset the procedure was as follows. First, a web-crawler was prepared based on Spatie’s crawler (https://github.com/spatie/crawler). Second, a list of hyperlinks of news sites in Spanish was compiled. As not all digital newspapers were focused solely on economy, two strategies were devised to identify which news items were showing economic content. On the one hand, we filter by url, as many websites post financial content in specific subsections (*e.g*., “https://www.elconfidencial.com/mercados/”). On the other hand, we also filter the content by using Cascading Style Sheets (CSS) and xPath (https://www.w3.org/TR/xpath-30/) rules, as some of the financial-related news items contain specific CSS classes. Besides, these filters allow the identification of the parts of the HTML files that contain the main headlines to, subsequently, discard those with content out of the scope of this work. Third, once the relevant web pages and news items have been identified, the full content of the article was downloaded transforming the HTML content into markdown. Finally, the first line of each document that contains the heading (*i.e*., the # symbol) is extracted. This split is composed by 2,266 headlines.

Both datasets were merged in the Spanish FinancES-TSA 2022 *corpus*, which is composed of a total of 3,829 documents. Table 1 depicts the main statistics of the full *corpus* and organized by data source. As it can be observed, the dataset is unbalanced for the MET, with few neutral emotions. However, the presence of neutral sentiments is more significant on the other two targets. The imbalance is noticeable in both data sources and it led to the choice of the Macro-F1 score as the metric in our sentiment classification experiments. The *corpus* has been made available for the scientific community (Rawdata2022.rar). The *corpus* contains an internal identifier, the text, the MET, the sentiment towards each considered target (*i.e*., MET, other companies, and society in general), the data source (either Twitter or news headlines), and the split used in this experiment (that is, whether the document was used during training, validation or testing).

**Table 1:** Summary of the Spanish FinancES-TSA 2022 *corpus* including the label balance for each target.

Dataset	MET			Society			Others			Total
	POS	NEU	NEG	POS	NEU	NEG	POS	NEU	NEG	TOTAL
Tweets	994	47	522	624	488	451	201	1,092	270	1,563
Headlines	1,436	139	691	807	1,035	424	549	1,328	389	2,266
Total	2,430	186	1,213	1,431	1,523	875	750	2,420	659	3,829

Next, the annotation process of the MET and the sentiment polarities for (i) the MET, (ii) other companies, and (iii) the society in general, is described. Each text was annotated by two of the authors of this work. The documents with disagreements were discussed and resolved by the other two authors. We prioritized to keep the shortest alternative for the MET, that is, the one that does not include any articles. However, in the cases where the article belongs to the name (*e.g*., “La Liga”^1^ 1The men’s top professional football division of the Spanish football league system.), they were kept. Besides, those documents in which there were two possible candidates for the MET that were far from each other in the text, were discarded. We kept, however, documents in which the MET is composed by two targets and a link (*e.g*., “Enagás y Elecnor”^2^ 2Two energy companies in Spain.) when both are associated to the same sentiment.

Table 2 contains examples of the *corpus*, including the text, the MET and the sentiment towards the considered targets. The first two examples are texts that are positive for the MET but negative to the rest. In the first example, it is stated that *‘Oil companies’ profits soar on higher crude oil prices’*. This fact is economically positive for oil companies but negative for other companies and for the society in general because higher crude oil prices result in more expensive raw materials and transportation. The second example states ‘*The Treasury will pocket 370 euros more per taxpayer in personal income tax due to inflation*’ and it is related to the fact that inflation will cause the government to collect more money from taxes, which would allow the state coffers to increase, and the government be able to apply the revenues in different types of policies. However, the short-term effects are that society has less money to invest, which will also have a negative impact on other companies. The third example is related to the Christmas campaign, that will generate new contracts which is positive, not only for the campaign, but for companies and citizens, as consumption and product offers are encouraged. The fourth example is related to a drop in the price of electricity during Christmas Eve which is negative for the electric power industry but positive to the rest of the companies and the rest of the society.

**Table 2:** Examples of the dataset, including the text in Spanish, the MET and the sentiment towards the MET (S. MET), other companies (S. Other companies) and society in general (S. Society).

	Text	MET	S. MET	S. other companies	S. society
1	Las petroleras disparan su beneficio por la subida del crudo	Petroleras	POS	NEG	NEG
2	Hacienda se embolsará 370 euros más por contribuyente en el IRPF gracias a la inflación	Hacienda	POS	NEG	NEG
3	La campaña de Navidad generará 453.260 contratos, un 5% más, según Randstad	campaña de Navidad	POS	POS	POS
4	El precio de la electricidad dará un leve respiro en Nochebuena	Precio de la electricidad	NEG	POS	POS

Finally, we split the dataset into training, validation of the hyperparameter optimization stage and testing in a 60-20-20 ratio for both the NER task and the SA module. It is worth noting that during our experiments we conduct an ablation analysis to analyze the performance of each dataset separately. In order to do a fair comparison between two data sources, the test split employed is the same, regardless of the data source.

### Preprocessing stage

The next step is a preprocessing stage, in which an alternative version of the texts is generated by removing hyperlinks and emoticons. Hashtags (*e.g*., #topic) and mentions (*e.g*., @name) have been kept because in some cases the MET is referenced from them.

### Main economic target detection

In this module of the architecture, the MET of a text is extracted. We rely on the same definition that it is given in the SemEval-2022 ‘Structured Sentiment Analysis’ shared task (Barnes et al., 2022), which indicates that the target is the receiver of a polarity expressed by a holder through a sentiment expression. Table 3 contains some examples of texts from the *corpus* and the corresponding MET that were manually annotated during *corpus* compilation.

**Table 3:** Examples of target identification, including the original text and the Main Economic Target (MET).

	Text	MET
1	Torrecid pone color español a la cerámica de los cinco continentes	Torrecid
2	Ventas de Gruma crecen 3%, registra aumentos de precios en México	Ventas de Gruma
3	Fidelity alcanza un 10% de Natra	Fidelity
4	Las bolsas del Sudeste Asiático comienzan con resultados mixtos	Bolsas del Sudeste Asiático
5	General Motors se dispara en Wall Street tras mejorar su previsión de beneficios	General motors

**Figure 2:**
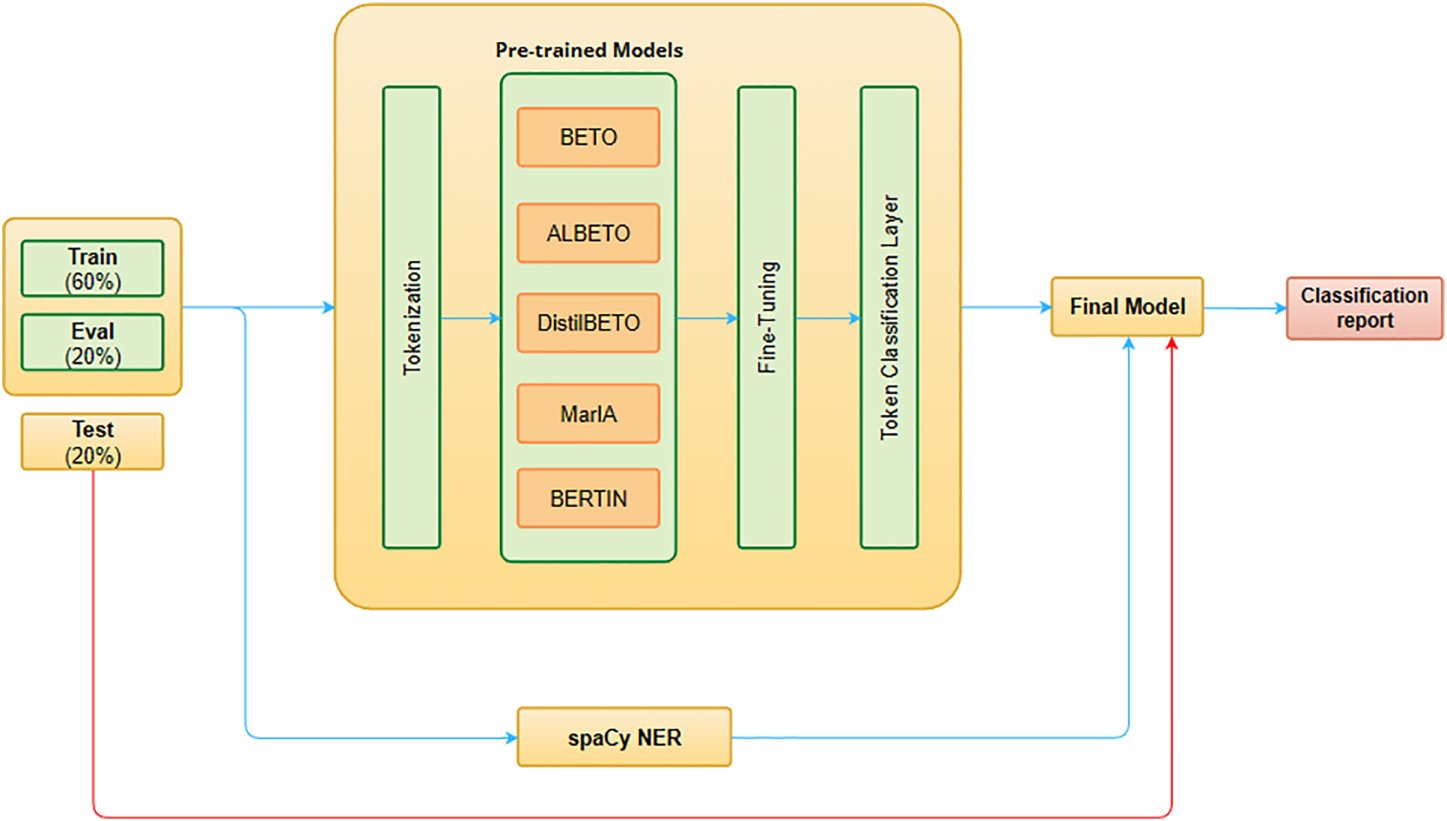
MET detection system architecture.

To automatically extract the MET of a text, several Spanish LLMs and existing NER frameworks are evaluated. We formulate the target identification problem as a sequence labeling task, in which the model predicts the entity for the input word tokens. To this end, two approaches have been explored (see Fig. 2).

The first method for extracting the MET involves customizing the NER model of the spaCy framework by training it with our *corpus*. SpaCy (https://spacy.io/) is one of the leading development frameworks in NLP model production that provides pre-trained models of different languages. The main reason spaCy has been preferred over other NLP frameworks for MET detection is that it offers a NER model that can recognize a wide range of entities including persons, organizations, languages, events, and others. In addition, spaCy provides the possibility to customize the model adding a new entity using your own training *corpus*. The main details of our approach are as follows. First, we apply a data processing step to transform the dataset into the specific format required for the training of the spaCy NER models. The training data must be formed by a list of tuples including the text and the initial and final position of the entities within the text. Moreover, the data must be in binary “.spacy” format. Second, we created an empty NER model in Spanish and trained it on the processed dataset.

The second approach for MET detection consists in creating new NER models by fine-tuning Spanish LLMs (see “Fine-Tuning”). Recent developments in transformer-based LLMs have proven successful in many NLP tasks across different languages. For Spanish, there are different monolingual models based on BERT (Devlin et al., 2019) or RoBERTa (Liu et al., 2019). However, these Spanish LLMs have been trained on various written text sources, such as the OPUS Project (Cañete et al., 2020) and the National Library of Spain (Gutiérrez-Fandiño et al., 2022), which are very distant from what we intend to address in sentiment prediction in the financial domain. The following models are evaluated:

- **BETO** (Cañete et al., 2020): It is a LLM based on BERT and pre-trained with data from Wikipedia and all the sources of the OPUS Project (Tiedemann, 2012) that have text in Spanish. The sources also include United Nations and Government journals, TED Talks, Subtitles, News Stories and more. BETO is similar in size to BERT and it was trained with the Whole Word Masking technique. According to Cañete et al. (2020), by fine-tuning this pre-trained model in Spanish, better results were obtained than other pre-trained BERT-based model on multilingual corpora for most tasks.
- **ALBETO** (Cañete et al., 2022): In recent years, considerable progress has been made with LLMs. Many approaches increase model size by pre-training natural language representations to improve performance on downstream tasks. But at some point, increasing the size of the model becomes more difficult due to GPU/TPU memory limitations and longer training times. Therefore, the inference time makes it impractical to use in real-world environments. To address this problem, ALBERT (Lan et al., 2020) presents two parameter-reduction techniques to lower memory consumption and increase the training speed of BERT. For this study, we have used ALBETO, a version of ALBERT pre-trained exclusively on Spanish *corpus*.
- **DistilBETO** (Cañete et al., 2022): DistilBERT (Sanh et al., 2019) is a small, fast, cheap, and light transformer model based on the BERT architecture. Knowledge distillation is performed during the pre-training phase to reduce the size of a BERT model by 40%. For this study, we have used a version of DistilBERT pre-trained exclusively on Spanish *corpus* called DistilBETO.
- **MarIA** (Gutiérrez-Fandiño et al., 2022): It is a Spanish LLM model based on RoBERTa. MarIA has been pre-trained using the largest Spanish *corpus* known to date, with a total of 570 GB of cleaned and deduplicated texts with 135 billion words extracted from the web crawling performed by the National Library of Spain from 2009 to 2019.
- **BERTIN** (de la Rosa et al., 2022): This model targets the best of all RoBERTa-based models trained from scratch on the Spanish part of mc4 dataset (Xue et al., 2021). This project is organized by the Flax Community (https://huggingface.co/flax-community). The pre-training of LLMs usually requires enormous amounts of computational and data resources. This model used the perplexity sampling technique to allow the pre-training of the model to be done in about half the number of steps and with one-fifth of the data normally needed.

For the MET detection task with fine-tuned Spanish LLMs, instead of spanning multiple entities like other existing NER models, we only include the target entity. Thus, using the IOB2 format (short for Inside, Outside, Beginning) for labeling tokens, there are a total of three entity classes for each word token: (i) B-TARGET, which indicates the beginning of the target entity, (ii) I-TARGET, which denotes that the token belongs to the target, and (iii) O, which signifies that the token does not belong to any target.

The main feature of LLMs based on transformers is their self-attention mechanism, whereby each word in the input is able to learn what relation it has with the others (Yi & Tao, 2019). All these models require the input data to be preprocessed through tokenization, which consists of decomposing a larger entity into smaller components called *tokens*. For this tokenization stage, specific tokenizers have been used for each model. Table 4 shows the type of tokenization used by each transformer model. All the models used in this study use the tokenization of sub-words, but with different algorithms, such as WordPiece for BETO and DistilBETO, SentencePiece for ALBETO, and Byte-level Byte-Pair Encoding (BPE) for the RoBERTa-based models (*i.e*., MarIA and BERTIN).

**Table 4:** Details of the models’ architecture. The tokenization used by each Transformer model is shown along with the number of layer (L), the number of hidden states of the model ( ${H_m}$), the dimension of the feed-forward layer ( ${H_{ff}}$), the number of attention heads (A), the size of the vocabulary (V), and the number of parameters (Params).

Model	Tokenization	L	A	${H_m}$	${H_{ff}}$	V	Params
BETO	WordPiece	12	12	768	3,072	31 K	110 M
ALBETO	SentencePiece	12	12	768	3,072	31 K	12 M
DistilBETO	WordPiece	6	12	768	3,072	31 K	67 M
MarIA	Byte-level BPE	12	12	768	3,072	50 K	125 M
BERTIN	Byte-level BPE	12	12	768	3,072	50 K	125 M

#### Fine-tuning

The fine-tuning stage consists of retraining a pre-trained LLM using the financial domain data. As a result of this process, the weights of the original model are updated to take into account the characteristics of the domain data and the token classification task. In our case, the fine-tune is done using a masked language model task (MLM), and then a token classification layer is added on the top of the LLM. For each model, we conduct an hyperparameter optimization stage to choose the best hyperparameters using RayTune (Liaw et al., 2018).

We evaluated 10 trials for each model and the following hyperparameters: (i) weight decay (between 0 and 0.3), (ii) training batch size ([8,16]), (iii) number of training epochs ([1–10]), and (iv) learning rate (between 1e−5 and 5e−5). Finally, the best models from each of the LLMs are evaluated on the test dataset.

### Sentiment classification methods

Targeted SA can provide more and deeper information than document-based SA, because it aims to predict the sentiment polarities of different targets in the same text. The main challenge of entity-based SA is that different entities, in our case the MET, other companies, and society in general, may have different polarities in the same text. In this article, we propose a targeted SA model based on different LLMs. Since three targets per text are considered, in this work two alternative approaches are tested and compared: (i) to have three distinct sentiment classification models, one per target; and (ii) to have a single multi-label classification model in which a label (either positive, negative or neutral) is produced for all three targets (*i.e*., the MET, other companies, and society in general).

**Figure 3:**
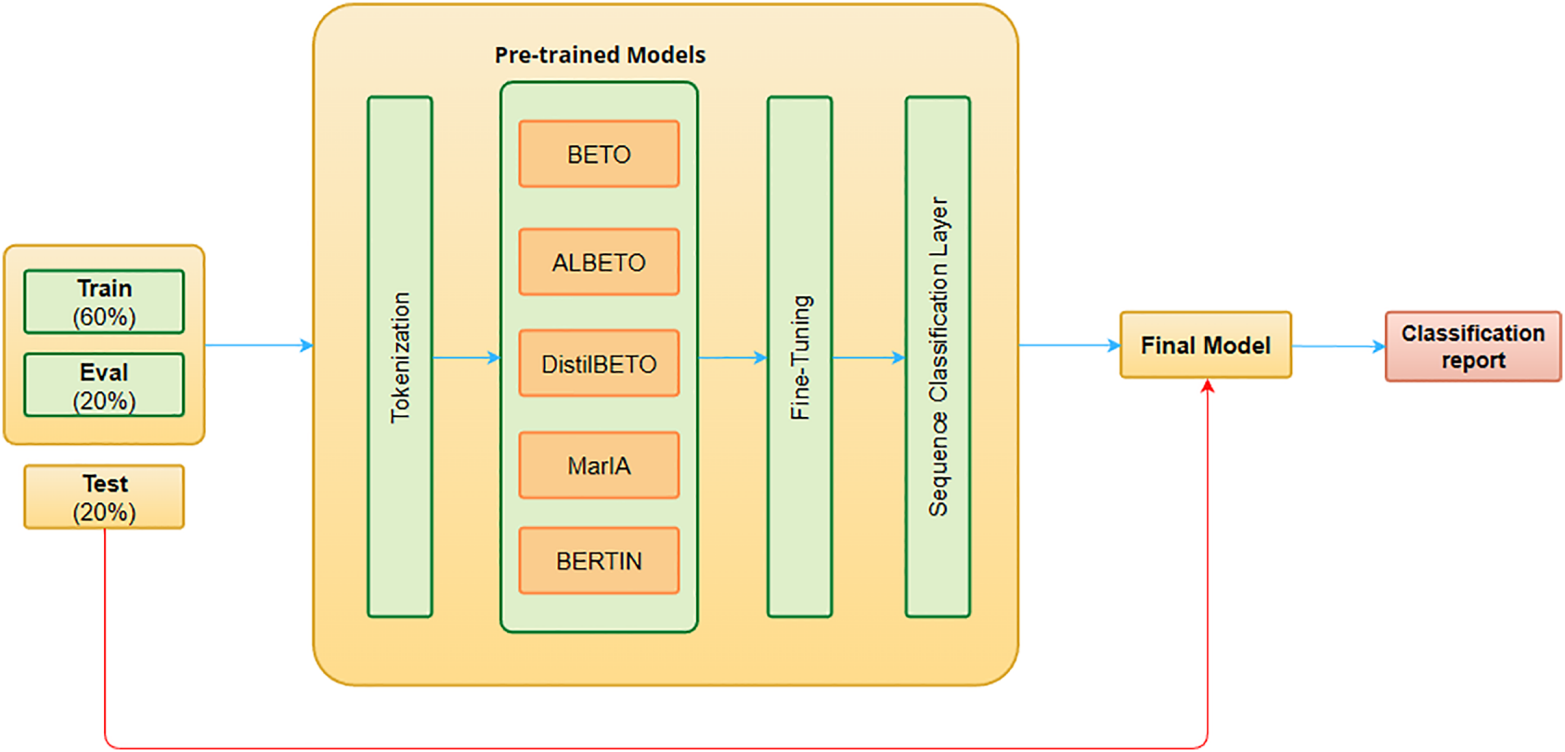
Sentiment classification system architecture.

In our first approach we consider each target as a different classification task, so we will have a sentiment classification model for each. Figure 3 shows the architecture of our proposal. Briefly, it can be described as follows.

1. The input is tokenized using the associated tokenizer of each LLM (see Table 4) to convert text strings into integer token IDs readable by transformer-based pre-trained models.
2. We use several LLMs as a starting point, as this reduces computational costs and allows us to use the latest generation models without having to train one from scratch.
3. LLMs are trained on a financial domain-specific dataset to fit a sequence classification task, transferring the knowledge from the pre-trained model. As in the case of the MET detection method, a hyperparameter optimization stage is carried out using RayTune to select the best combination of the hyperparameters over 10 trials. The hyperparameters evaluated and their range are: (i) weight decay (between 0 and 0.3), (ii) training batch size ([8,16]), (iii) number of training epochs ([1–10]), and (iv) learning rate (between 1e−5 and 5e−5).
4. Finally, we add a sequence classification layer on the top of the pre-trained models and the best models from each of the pre-trained models are evaluated on the test dataset.

The main issue with the above approach is that it uses normal classification models in which the class labels are mutually exclusive, *i.e*., for each target there can only be one sentiment. Therefore, it is necessary to create a sentiment classification model for each target, which can be very computationally expensive and difficult to implement in real scenarios. For this reason, we compare the results obtained with the previous method against the results using a single multi-label classification model that outputs a distinct sentiment for each considered target. The architecture of this approach is the same as the previous one (see Fig. 3), but it is necessary to convert the single-label dataset into a multi-label dataset. To do this, we use the one-hot representation, which consists of creating different columns according to the possible labels and assigning boolean values to them.

## Results and analysis

In this section, we report and discuss the results of the best model with the test split both for the MET detection models (see “Results of main economic target detection”) and for the sentiment classification models (see “Results of sentiment classification models”). All results are reported both with the complete dataset and with the splits composed by only tweets or only headlines.

Since we address an imbalanced classification problem, we evaluated the performance of the deep learning models with macro-average F1-score and weighted-average F1-score. Both metrics measure of the performance of a classification model in terms of precision and recall. However, they differ in how they are calculated and how they weight the contributions of each class. The macro-average F1-score is calculated by taking the average of the F1 score for each class. This means that each class is given an equal weight in the calculation and gives information about the performance of the model with respect to each class independently. The weighted-average F1-score, on the other hand, takes into account the number of instances in each class, giving more weight to the classes with more instances. Thus, it measures the overall performance of the model in an unbalanced dataset.

### Results of main economic target detection

This section discusses the results obtained with the two approaches for the extraction of the MET mentioned above (see “Main economic target detection”). First, the results obtained in the hyperparameter optimization stage are scrutinized. In Table 5, the best subset of hyperparameters obtained for each LLM is shown. It can be observed that most of the models, including BETO, ALBETO and BERTIN, have obtained better results when using a large epoch and a training batch size of 16. However, the DistilBETO model works better with a smaller training batch size and with a larger weight decay. Second, the proposed approaches for MET detection were evaluated using the test split. The performance of the weighted version of precision, recall, and F1-score for the MET model have been considered. For the LLMs, the models are trained using their best subset of hyperparameters.

**Table 5:** Best subset of hyperparameters for each LLM for the MET detection model.

Model	Epoch	Weight decay	Batch size	Learning rate
BETO	10	0.0448468569	16	0.000034
ALBETO	10	0.0139110539	16	0.000039
DistilBETO	10	0.1950135486	8	0.000028
MarIA	7	0.0734046361	16	0.000038
BERTIN	8	0.0139110539	16	0.000039

In Table 6, the results obtained for the detection of MET with the two approaches are presented. It can be observed that LLMs perform better than spaCy. Besides, we found a difference in performance between the two main splits of the whole *corpus*. On the one hand, regarding the financial tweets split, all the LLMs, except ALBETO, perform better than spaCy. It is worth noting that ALBETO is a lighter model trained with few parameters. As for the headlines split and the complete dataset, the behavior is exactly the same as with the financial tweets split, with the BETO model remaining the best of all with a F1-score of 0.6560 and ALBETO still being the worst. The benefits of training with the full *corpus*, since it is more generic and varied, are reflected in the results. The best MET extraction approach with the complete *corpus* is BETO, with a Macro-F1 of 0.69, which is almost 4% better than the model with the split composed only by financial headlines and 8% better than the split composed only by financial tweets. Comparing BETO with its lightweight alternatives, ALBETO and DistilBETO, we observe a drop in performance of 6.67% for ALBETO and 5.24% for DistilBETO. Concerning the models based on RoBERTa (MarIA and BERTIN), we notice that spaCy achieves better results than BERTIN (0.77% of improvement) but not than MarIA (−1.29% of deterioration).

**Table 6:** Performance of MET detection model using different LLMs and spaCy.

Dataset	Model	Precision	Recall	F1-score
Tweets	BETO	0.632682	0.603598	0.617797
	ALBETO	0.528998	0.561043	0.54455
	DistilBETO	0.571313	0.585950	0.578539
	MarIA	0.551669	0.586644	0.568619
	BERTIN	0.596184	0.589159	0.592651
	spaCy	0.6099071207	0.5130208333	0.5572842999
Headlines	BETO	0.6452513	0.667148	0.656017
	ALBETO	0.571177	0.6114769	0.590640
	DistilBETO	0.604351	0.616269	0.610252
	MarIA	0.651957	0.622416	0.636844
	BERTIN	0.624006	0.608056	0.615928
	spaCy	0.6363636364	0.5651041667	0.5986206897
Total	BETO	0.701815	0.693103	0.697432
	ALBETO	0.632689	0.628821	0.630749
	DistilBETO	0.639000	0.651067	0.644978
	MarIA	0.666136	0.670400	0.668261
	BERTIN	0.651033	0.644374	0.647687
	spaCy	0.7108066971	0.6080729167	0.6554385965

Finally, we analyzed the predictions obtained with the best model, namely, the fine-tuned BETO model with the full *corpus*. We found that in some of the texts where the MET is not so explicit, the model identifies all possible entities, as shown in texts one, two and three of Table 7. On the other hand, we also observed that when the MET is composed of several words, the model sometimes fails to predict the whole MET, leaving some words out, as in the case of texts four and five present in Table 7.

**Table 7:** Results of error analysis of BETO trained with the whole *corpus* for MET detection on five misclassified examples from the test dataset.

	Text	MET	Predict
1	Competencia del Reino Unido denuncia la compra de Giphy por parte de Facebook, ya que podría perjudicar a usuarios y anunciantes.	Facebook	Giphy, Facebook
2	El mayor error que puede cometer un emprendedor ahora mismo, según el cofundador de Let’s Bonus y Wallapop	un emprendedor	emprendedor, Let’s Bonus
3	Industria concede 54 millones a Pachá, Eating Group, Cesgarden y Egile para recuperarse de la covid-19	Pachá, Eating Group, Cesgarden y Egile	Pachá, Eating Group, Cesgarden
4	El sector comercial afronta la campaña de Navidad con menos personal que antes de la pandemia	El sector comercial	Sector comercial
5	El sector de las agencias de viaje, que esperaba recuperar este verano hasta un 40% de la facturación de 2019, prevé ingresar sólo entre el 25% y el 30%.	El sector de las agencias de viaje	Sector de las agencias de viaje

### Results of sentiment classification models

For the sentiment classification models, the Macro-F1 score is used to compare the performance of the different LLMs examined. The main reason for using the Macro-F1 metric is that the datasets are unbalanced, as shown in “Datasets”, and this measure would give equal weight to all labels, even if some have fewer examples.

We evaluated the proposed LLMs both using the whole *corpus* and just the splits composed by only tweets or only headlines. Using the model architecture depicted in Fig. 3, we fine-tuned all LLMs using the best subset of hyperparameters obtained during the hyperparameter optimization stage, which are shown in Table 8. The hyperparameters vary a lot between the target entities, since within the same text, sentiment may change depending on the target. However, BETO and MarIA models always perform better with a training batch size of 16 and ALBETO with a training batch size of 8.

**Table 8:** Best subset of hyperparameters for each model and each target entity.

Target entity	LLM	Epochs	Batch size	Weight decay	Learning rate
MET	BETO	4	16	0.044847	0.000034
	ALBETO	8	8	0.011343	0.000022
	DistilBETO	4	16	0.073405	0.000038
	MarIA	8	16	0.188325	0.000017
	BERTIN	6	8	0.081163	0.000017
Society	BETO	9	16	0.073405	0.000038
	ALBETO	7	8	0.195014	0.000028
	DistilBETO	10	16	0.073405	0.000038
	MarIA	6	16	0.188325	0.000017
	BERTIN	7	16	0.126252	0.000012
Others companies	BETO	9	16	0.044847	0.000034
	ALBETO	7	8	0.011343	0.000022
	DistilBETO	7	8	0.011343	0.000022
	MarIA	8	16	0.073405	0.000038
	BERTIN	9	8	0.195014	0.000028

The results for the sentiment classification models built for each individual target are discussed in “Sentiment towards main economic target”, “Sentiment towards society” and “Sentiment towards other companies”, to then focus on the results of the multi-label sentiment classification approach in “Sentiments towards all targets using a multi-label sentiment classification approach”.

#### Sentiment towards main economic target

Table 9 shows the results of the sentiment classification models towards the MET. It can be noticed that the best result is obtained by the MarIA model with the full *corpus*, with a Macro-F1 of 75.97% and an overall performance of 85.88%. When comparing the results obtained with the different LLMs, it is possible to observe that the performance improves progressively as one evolves towards a more powerful and complex LLM, as in the case of MarIA, which is the model with the largest training *corpus* based on RoBERTa. Furthermore, with the split composed of financial tweets, there are several models that have scored 0 in detecting neutral sentiment towards the MET, such BERTIN, ALBETO, and DistilBETO (not shown in the table). This is due to an underfitting problem, as there are very few tweets with neutral sentiment towards the MET in the training set as pointed out in “Datasets”. However, the weighted precision, recall, and F1 score metrics of these models are still quite high, because the average result that accounted for these metrics is the contribution of each class weighted by the number of examples of that given class. Moreover, it has been observed that ALBETO, despite being a lightweight model with the smallest number of parameters as shown in Table 4, has performed better than other more complex models such as BERTIN and BETO.

**Table 9:** Benchmark of the different LLMs with the whole *corpus* and the two splits evaluated for sentiment towards the MET. For each model and dataset, the weighted precision (W-P), weighted recall (W-R), weighted (W-F1) and macro (M-F1) are reported.

Dataset	Model	W-P	W-R	W-F1	M-F1
Tweet	BETO	0.8227	0.8266	0.8193	0.6284
	ALBETO	0.7813	0.8110	0.7950	0.5386
	DistilBETO	0.7652	0.7992	0.7818	0.5268
	MarIA	0.8200	0.8292	0.8238	0.6371
	BERTIN	0.7884	0.8120	0.7999	0.5409
Headlines	BETO	0.8336	0.8409	0.8341	0.6596
	ALBETO	0.8034	0.8031	0.8019	0.6629
	DistilBETO	0.7945	0.8018	0.7967	0.6214
	MarIA	0.8380	0.8370	0.8375	0.6949
	BERTIN	0.8006	0.8031	0.8018	0.6564
Total	BETO	0.8428	0.8422	0.8424	0.7259
	ALBETO	0.8428	0.8435	0.8428	0.7336
	DistilBETO	0.8068	0.8136	0.8089	0.6599
	MarIA	0.8580	0.8605	0.8588	0.7597
	BERTIN	0.8270	0.8201	0.8229	0.6743

In all, different LLMs with various characteristics have been evaluated. There are models that behave well in some cases but not so well in others. In particular, there are many cases where the best model, namely, MarIA with the full *corpus*, does not predict correctly while other models do. For this reason, different ensemble learning strategies have been tested on the models trained with the full *corpus* to see if the models complement each other and improve the classification results. In ensemble learning the output of each model trained with a set of text features is combined by either averaging the prediction (mean), calculating the mode of the predictions (mode), or choosing the prediction with the highest probability (max). Table 10 shows the results of these different ensemble learning techniques. The mode-based ensemble learning strategy has archived a 0.07% improvement in Macro-F1 over MarIA, which is the best model of the complete *corpus*.

**Table 10:** Benchmark of different ensemble learning strategies with the whole *corpus* evaluated for sentiment towards the MET. Weighted precision (W-P), weighted recall (W-R), weighted (W-F1) and macro (M-F1) are reported.

	W-P	W-R	W-F1	M-F1
Max	0.8496	0.8527	0.8484	0.7419
Mean	0.8575	0.8605	0.8583	0.7548
Mode	0.8547	0.8566	0.8543	0.7604

**Figure 4:**
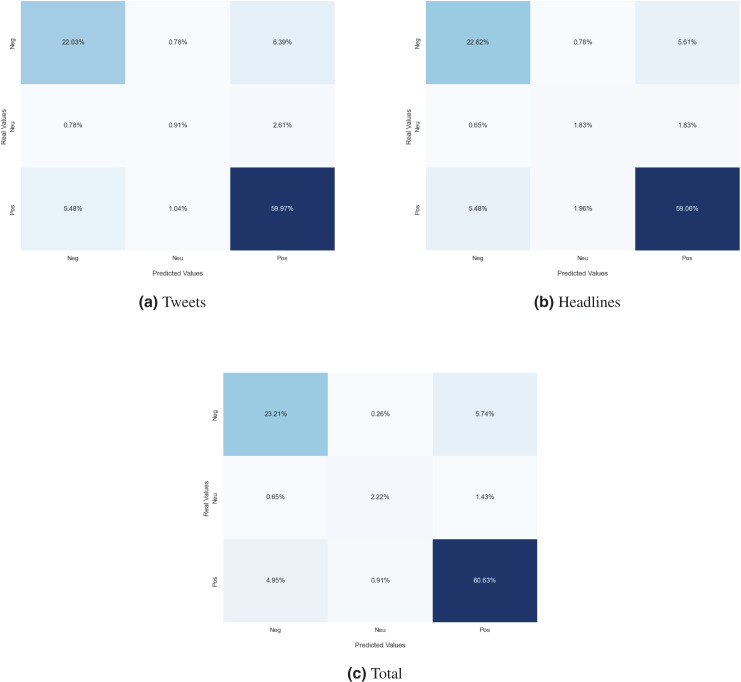
Confusion matrix of the MarIA model evaluated for sentiment towards the MET trained with financial tweets (A), financial news headlines (B) and the whole *corpus* (C).

Next, we analyzed the limitations of these models in predicting the sentiment polarity towards the MET. For this purpose, the confusion matrix has been used to evaluate the examples misclassified by the best classification model for each dataset. The confusion matrix indicates, for each sentiment class, the distribution of predictions made by the classification model. The diagonal of the matrix contains the proportion of correctly recognized sentiment instances, while off-diagonal elements denote the proportion of sentiment instances that have been wrongly classified. In Fig. 4, the confusion matrix of the best model for each dataset is represented. Concerning the financial tweets model (Fig. 4A), a large proportion of neutral instances are misclassified as either positive or negative (“neu-pos” and “neu-neg”). This is mainly due to an under fitting problem, as there are not enough training samples in the case of the neutral class. This behavior can be also observed with the headlines split (Fig. 4B) but not in the complete dataset (see Fig. 4C), which suggests that as the number of training samples increases, the number of these errors decreases.

On the other hand, the “pos-neg” and “neg-pos” categories are also among the most frequent errors. To analyze these cases in detail, ten random examples have been examined in which all LLMs trained with the whole *corpus* predicted wrongly the sentiment polarity towards the MET. The results are shown in Table 11. After analyzing these erroneous cases, we found out that all LLMs produce the same output when the text contains words with negative or positive polarity, such as for example in Text 1 with the word “aumentó”, which has a positive polarity, but the text expresses a negative sentiment for the target. The same behavior is observed in the remaining examples. Through error analysis, we can see that, although current models have made significant progress, there are still some controversial sentences that are beyond the capabilities of these models. These limitations might be caused by the lack of context knowledge about the entities. If that is the case, it would be necessary to evaluate these models with longer texts and with models trained on economic texts.

**Table 11:** Results of error analysis of the LLMs trained with the whole *corpus* on 10 misclassified examples from the test dataset. The misleading words that cause the error are in bold.

	Text	BETO	BERTIN	MarIA	ALBETO	DistilBETO	Label
1	el precio medio de la vivienda **aumentó** el 5,5% en 2018, según la tasadora st	pos	pos	pos	pos	pos	neg
2	nike esquiva **la decepción**, pero su futuro sigue pendiente de china	pos	pos	pos	pos	pos	neg
3	urgente—la eurozona se **frenó** el cuarto trimestre y registró un crecimiento del 5,2% en 2021	neg	neg	neg	neg	neg	pos
4	la bolsa española **reparte** su aguinaldo con el pago de 16 dividendos hasta reyes, por orespain	pos	pos	pos	pos	pos	neu
5	hacienda coloca en su punto de mira la compra de criptomonedas con tarjeta	pos	pos	pos	pos	pos	neg
6	las **bajas** del mobile world congress	neg	neg	neg	neg	neg	neu
7	la vivienda nueva y usada **subió** un 7,5% en diciembre de 2021	pos	pos	pos	pos	pos	neg
8	el precio medio de la vivienda **aumentó** el 5,5% en 2018, según la tasadora st	pos	pos	pos	pos	pos	neg
9	el precio de la vivienda nueva **sube** un 5% en 2017, la mayor subida en diez años	pos	pos	pos	pos	pos	neg
10	los carburantes repuntan a nuevos **máximos** y acumulan un **encarecimiento** de hasta el 15%	pos	pos	pos	pos	pos	neg

#### Sentiment towards society

The results obtained from the different sentiment classification models for the society target are shown in Table 12. Again, it can be observed that using the whole *corpus* leads to a better outcome. Comparing the results obtained with the different pre-trained models, once more MarIA achieves the best results with a Macro-F1 of 74.16% and an overall performance of 74.06%.

**Table 12:** Benchmark of the different LLMs with the whole *corpus* and the two splits evaluated for sentiment towards society in general. For each model and dataset, the weighted precision (W-P), weighted recall (W-R), weighted (W-F1) and macro (M-F1) are reported.

Dataset	Model	W-P	W-R	W-F1	M-F1
Tweets	BETO	0.6869	0.6858	0.6852	0.6860
	ALBETO	0.6445	0.6428	0.6413	0.6382
	DistilBETO	0.6540	0.6532	0.6530	0.6499
	MarIA	0.6887	0.6884	0.6853	0.6878
	BERTIN	0.6314	0.6310	0.6290	0.6258
Headlines	BETO	0.6725	0.6688	0.6675	0.6617
	ALBETO	0.6130	0.6128	0.6110	0.6050
	DistilBETO	0.6332	0.6284	0.6250	0.6154
	MarIA	0.6820	0.6701	0.6701	0.6717
	BERTIN	0.6116	0.6115	0.6041	0.5868
Total	BETO	0.7304	0.7288	0.7286	0.7286
	ALBETO	0.6439	0.6428	0.6422	0.6385
	DistilBETO	0.6688	0.6688	0.6686	0.6659
	MarIA	0.7419	0.7405	0.7406	0.7416
	BERTIN	0.6976	0.6975	0.6964	0.6885

Besides, we have also evaluated different ensemble learning strategies on the models trained with the whole *corpus* to check if the combination of the models’ outputs resulted in improved performance. The results of the different ensemble learning techniques explored are shown in Table 13. It can be observed that no improvement is accomplished in the classification results, which leads us to believe that the models do not complement each other in this case.

**Table 13:** Benchmark of different ensemble learning strategies with the whole *corpus* evaluated for sentiment towards the society. Weighted precision (W-P), weighted recall (W-R), weighted (W-F1) and macro (M-F1) are reported.

	W-P	W-R	W-F1	M-F1
Max	0.7246	0.7236	0.7231	0.7221
Mean	0.7373	0.7366	0.7366	0.7375
Mode	0.7319	0.7314	0.7315	0.7330

**Figure 5:**
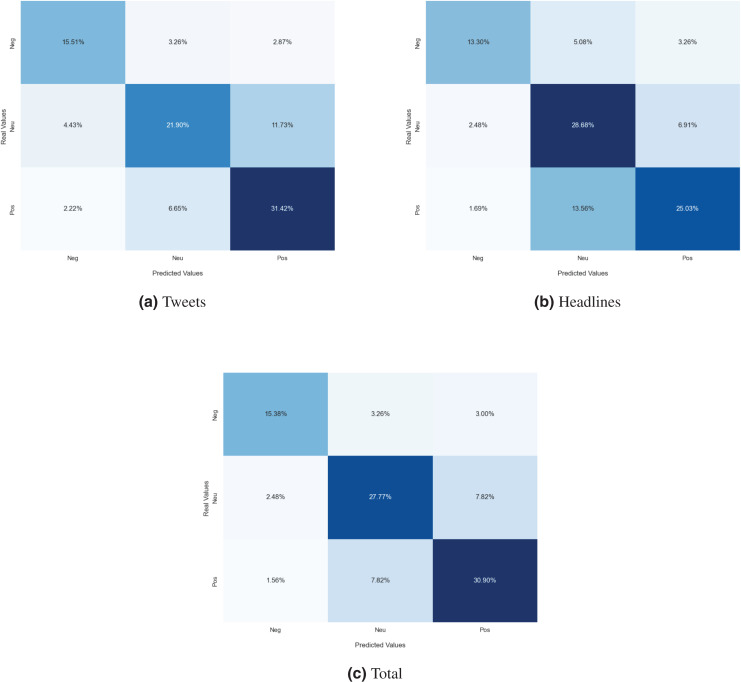
Confusion matrix of the MarIA model evaluated for sentiment towards society trained with financial tweets (A), financial news headlines (B) and the whole *corpus* (C).

In the case of the sentiment towards society in general, the distribution of polarities is balanced, so both weighted and macro metrics are similar and there is not a big difference. The confusion matrix of the best model for each dataset is depicted in Fig. 5. In the figure it is possible to observe which types of sentiments are more confused with others. In the set of financial tweets (see Fig. 5A), positive sentiment is confused with neutral sentiment (“pos-neu”) with 11.73% and 6.65% the other way around (“neu-pos”). Many types of errors, such as neutral and positive instances misclassified as negative (“neu-neg”, “pos-neg”), are less likely as the dataset is larger and more generic, as is the case for the financial news headlines split (see Fig. 5B) and the whole *corpus* (see Fig. 5C).

#### Sentiment towards other companies

In this section, we evaluate the performance of the sentiment classification models for the target ‘other companies’. The results are shown in Table 14. Similarly to the previous cases, the best results are reached when training the models with the whole *corpus*, since it constitutes a more generic training set. With respect to the pre-trained models, BETO is the one that has obtained the best result, with a Macro-F1 of 67.11% and an overall performance of 74%. It has also been observed that the BERTIN model has performed worse despite having a more complex architecture and more vocabulary size than other models such as BETO, ALBETO and DistilBETO.

**Table 14:** Benchmark of the different LLMs with the whole *corpus* and the two splits evaluated for sentiment towards other companies. For each model and dataset, the weighted precision (W-P), weighted recall (W-R), weighted (W-F1) and macro (M-F1) are reported.

Dataset	Model	W-P	W-R	W-F1	M-F1
Tweets	BETO	0.7161	0.7288	0.7031	0.6056
	ALBETO	0.6532	0.6806	0.6600	0.5448
	DistilBETO	0.6765	0.7001	0.6757	0.5730
	MarIA	0.7088	0.7158	0.7049	0.6144
	BERTIN	0.6184	0.6441	0.6271	0.5055
Headlines	BETO	0.6832	0.6728	0.6770	0.6035
	ALBETO	0.6541	0.6375	0.6438	0.5657
	DistilBETO	0.6453	0.6375	0.6409	0.5491
	MarIA	0.6858	0.6780	0.6809	0.6054
	BERTIN	0.6569	0.5698	0.5924	0.5109
Total	BETO	0.7384	0.7445	0.7400	0.6711
	ALBETO	0.7251	0.7327	0.7280	0.6503
	DistilBETO	0.7155	0.7223	0.7177	0.6352
	MarIA	0.7373	0.7445	0.7382	0.6655
	BERTIN	0.7020	0.6741	0.6816	0.6028

In addition, we also check whether combining the results of all models trained with the whole *corpus* using different ensemble learning techniques improves the classification results. In this case, mean-based ensemble learning has managed to improve by up to 0.93% in Macro-F1 of the best model (*i.e*., BETO), as shown in Table 15.

**Table 15:** Benchmark of different ensemble learning strategies with the whole *corpus* evaluated for sentiment towards the other companies. Weighted precision (W-P), weighted recall (W-R), weighted (W-F1) and macro (M-F1) are reported.

	W-P	W-R	W-F1	M-F1
Max	0.7487	0.7536	0.7445	0.6726
Mean	0.7517	0.7601	0.7529	0.6807
Mode	0.7413	0.7497	0.7426	0.6708

**Figure 6:**
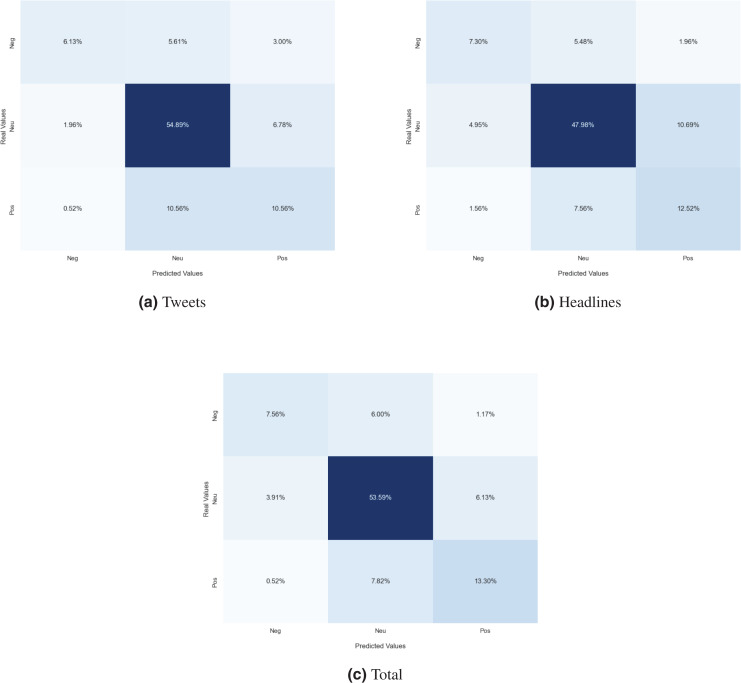
Confusion matrix of the best model evaluated for sentiment towards other companies trained with financial tweets (A), financial news headlines (B) and the whole *corpus* (C).

In the case of the sentiment towards other companies, the distribution of the polarities of the three sentiments is unbalanced. As pointed out in “Datasets”, the number of examples of the neutral sentiment is much higher than that of the other two. Therefore, there is a notable difference in the values of the W-F1 and M-F1 metrics; the weighted metrics, since the number of examples of each sentiment are not taken into account, tend to obtain better results than Macro-F1. Analyzing the confusion matrices of the best model of each dataset (see Fig. 6), we can see that exactly the same behavior as with the society target is exhibited: positive instances misclassified as neutral (“pos-neu”) and neutral instances misclassified as positive (“neu-pos”) are the most frequent errors and the error rate decreases as the model is trained with a larger and more varied training set.

#### Sentiments towards all targets using a multi-label sentiment classification approach

The main problem with the previous sentiment classification models in this multi-target environment for the financial domain is that a model is needed for each possible target. To overcome this limitation, a multi-label approach is evaluated in which a single multi-label model is able to predict the sentiment of different targets. In order to obtain the best result for each of the considered LLMs, a hyperparameter optimization process has been performed to obtain the best subset of hyperparameters for each model. In Table 16 the hyperparameters that led to the best results for each pre-trained model are listed. In this case, all models work better with a higher training epoch and most of them with a training batch size of 8, except for DistilBETO, which works best with a batch size of 16.

**Table 16:** Best subset of hyperparameters for each model in multi-label sentiment classification.

Model	Epoch	Batch size	Weight decay	Learning rate
BETO	10	8	0.195014	0.000028
ALBETO	8	8	0.039639	0.000028
DistilBETO	9	16	0.073405	0.000038
MarIA	10	8	0.195014	0.000028
BERTIN	10	8	0.081163	0.000017

The results of the experiment are shown in Table 17. Again, training the models with the whole *corpus*, more generic and varied, produces the best results. The best pre-trained model is BETO, with a Macro-F1 of 71.13% and an overall performance of 76.80%. We can see that this result is slightly worse than the sentiment classification models built for the MET (see “Sentiment towards main economic target”) and the society target (see “Sentiment towards society”), and slightly better than the models built for the ‘other companies’ target (see “Sentiment towards other companies”).

**Table 17:** Benchmark of the different LLMs with the whole *corpus* and the two splits evaluated for multi-label sentiment classification. For each model and dataset, weighted precision (W-P), weighted recall (W-R), weighted (W-F1) and macro (M-F1) are reported.

Dataset	Model	W-P	W-R	W-F1	M-F1
Tweets	BETO	0.7476	0.7362	0.7314	0.6397
	ALBETO	0.7170	0.6997	0.6928	0.5664
	DistilBETO	0.7060	0.7058	0.6971	0.5729
	MarIA	0.7669	0.7445	0.7395	0.6398
	BERTIN	0.7421	0.7171	0.7102	0.6079
Headlines	BETO	0.7400	0.7253	0.7284	0.6515
	ALBETO	0.7137	0.7006	0.7021	0.6133
	DistilBETO	0.6973	0.6710	0.6808	0.5892
	MarIA	0.7439	0.7266	0.7301	0.6542
	BERTIN	0.7301	0.7188	0.7165	0.6171
Total	BETO	0.7734	0.7640	0.7680	0.7113
	ALBETO	0.7399	0.7332	0.7324	0.6543
	DistilBETO	0.7468	0.7279	0.7338	0.6561
	MarIA	0.7827	0.7597	0.7678	0.7017
	BERTIN	0.7600	0.7553	0.7516	0.6679

Finally, we analyze the limitations of the multi-label approach to predict the sentiment of different targets in the same text. For this purpose, we use the best model obtained, namely, BETO trained with the whole *corpus*, and scrutinize in detail the performance of the model. As it can be observed in Table 18, the model experiences problems in detecting the neutral sentiment of the MET and the positive sentiment of the ‘other companies’ target. This might be due to the scarce number of samples of this kind in the training set. For this reason, the model often confuses neutral sentiment towards the MET with positive or negative sentiment, and is sometimes unable to predict positive sentiment towards the target ‘other companies’, as shown in the examples of Table 19.

**Table 18:** Performance of BETO with the full *corpus* evaluated for multi-label sentiment classification.

	Precision	Recall	F1-score
**MET**			
Positive	0.8806	0.8824	0.8815
Neutral	0.7000	0.4242	0.5283
Negative	0.7555	0.7723	0.7638
**Other companies**			
Positive	0.6301	0.5542	0.5897
Neutral	0.8187	0.8238	0.8212
Negative	0.6174	0.6283	0.6228
**Society**			
Positive	0.7442	0.7249	0.7344
Neutral	0.7314	0.7089	0.7200
Negative	0.7267	0.7530	0.7396
**Combined**			
**Micro avg**	0.7751	0.7640	0.7695
**Macro avg**	0.7339	0.6969	0.7113
**Weighted avg**	0.7734	0.7640	0.7680

**Table 19:** Results of error analysis of BETO trained with the whole *corpus* on six misclassified examples from the test dataset. Sentiment towards the MET is annotated as “target_pos”, “target_neu” or “target_neg”, towards other companies as “other_pos”, “other_neu” or “other_neg” and towards society in general as “society_pos”, “society_neu” or “society_neg”.

	Text	Label	Predict
1	UnicajaBanco ha planteado su propuesta de reducir su plantilla en 1.513 empleados, de los que 1.005 pertenecen a la red de oficinas y 508 a servicios centrales.	[target_neu, other_neu, society_neg]	[target_pos, other_neu, society_pos]
2	Horos refuerza su apuesta por carbón y petróleo ante una “acelerada y desordenada” transición energética	[target_neu, other_neu, society_neu]	[target_pos, other_neu, society_pos]
3	El BCE ligará los dividendos de la banca a los test de estrés	[target_neu, other_neu, society_neu]	[target_pos, other_neu, society_pos]
4	Rentabilidad y compromiso social, motivos para la inversión sostenible	[target_pos, other_pos, society_pos]	[target_pos, society_pos]
5	RSC.- Fundación ‘Integra’ pide “concienciación” a los responsables de RRHH de las empresas en los procesos de selección	[target_pos, other_pos, society_pos]	[target_pos, society_pos]
6	Desde la moncloa avanza cambios en la tarifa regulada (PVPC) para tratar de estabilizar la factura de la luz y descarta una intervención pública del mercado.	[target_pos, other_pos, society_pos]	[target_pos, other_neg, society_pos]

## Conclusions and further work

In this work, a novel *corpus* has been compiled for financial targeted sentiment classification. Two data sources have been considered, namely, Twitter and newspapers headlines. Besides, the *corpus* has been manually annotated identifying the MET and the sentiment of each text towards three disparate targets: the MET, other companies and society in general. The aforementioned *corpus* is used to train and evaluate a Financial Targeted Sentiment Analysis system based on state-of-the-art Spanish LLMs including BETO, ALBETO, DistilBETO, MarIA and BERTIN. The results for the MET extraction stage and for the sentiment classification task has been analyzed separately. Besides, in our rigorous analysis the models have been evaluated both using the whole *corpus* and each separated split, one composed only by tweets and the other only by news headlines. Moreover, two approaches for the multi-target sentiment analysis endeavor have been explored: the use of a separated classification model for each target and the use of a multi-label technique in which only one model needs to be trained. The dataset (Rawdata2022.rar) and source code are publicly available.

We have observed that training the models with the whole *corpus* leads to better results. Concerning the evaluated Spanish LLMs, MarIA and BETO are the ones that have obtained the best results. Our best result has a performance of 69.74% for the MET detection, and an overall performance of 76.04%, 74.16%, and 68.07% for the sentiment classification of the MET, society, and other companies target, respectively. The performance of the multi-label classification models in this scenario is slightly worse with an overall accuracy of 71.13%. We have also reached the following insights:

- The fine-tuning approach of LLMs for NER models is better than training the spaCy NER model from scratch.
- The fine-tuning approach of LLMs has given good results in SA for different targets, despite the fact that the training set is not very large.
- It is possible to apply a multi-label approach for targeted SA to extract the sentiment for all targets at the same time; but the overall performance is slightly lower than the one of a target-by-target SA approach.
- Lightweight models such as ALBETO and DistilBETO perform worse in our system compared to other models trained with a large *corpus* such as MarIA and BETO.
- The analysis of the two splits of the whole *corpus* (financial tweets and news headlines) resulted in a poorer performance as compared with use of the whole *corpus*. This suggests that each split complement each other. The complete *corpus* has improved the results of the MET detection, and the SA of each target.
- The use of ensemble learning combining different LLMs is beneficial *vs*. the use of each model separately. This indicates that the models are complementary. However, there are cases where the worst prediction dominates the rest of predictions, which worsens the performance of the best pre-trained model, as in the case of the SA for society target.

As future work, we will extend the *corpus* to improve the performance of sentiment classification and MET detection models. For this, we will use the UMUCorpusClassifier tool (García-Díaz et al., 2020) that simplifies the *corpus* annotation process with multiple annotators. On the other hand, while the experiments performed with the provided dataset obtained encouraging results, they are difficult to compare with other approaches. Consequently, we plan to adopt existing datasets for targeted sentiment analysis in different languages and for different application domains to enable a comparative analysis with related works (*e.g*., (Orbach et al., 2021; Hamborg & Donnay, 2021; Mutlu & Özgür, 2022)).

As promising future line of research, we also propose to investigate other frameworks such as Stanza, which is similar to spaCy and allows training to create a new NER model with our data and other models for target detection. Finally, we plan to develop a sentiment analysis system where the polarity of the comments of a financial tweet or financial news is determined to obtain a more accurate prediction about society’s sentiments and thus include it in the current model. Besides, we suggest measuring the relationship between financial targeted data and political ideology, as the polarities of data can be heavily biased according to psychographic traits of the citizens (García-Díaz, Colomo-Palacios & Valencia-García, 2022).

## Supplemental Information

10.7717/peerj-cs.1377/supp-1 Supplemental Information 1 Code.

10.7717/peerj-cs.1377/supp-2 Supplemental Information 2 Raw data.

## Additional Information and Declarations

### Data Availability

The following information was supplied regarding data availability: The raw data and code are available in the Supplemental Files.
